# Endocannabinoid system and skeletal muscle health: Insights from cannabidiol

**DOI:** 10.1016/j.molmet.2026.102396

**Published:** 2026-06-09

**Authors:** Anaïs Deglos, Nicolas Saroul, Stéphane Walrand, Olivier Le Bacquer

**Affiliations:** 1 Unité de Nutrition Humaine (UNH), Université Clermont Auvergne, INRAE, CRNH Auvergne, Clermont-Ferrand, France; 2 Service d'orl et chirurgie cervico-faciale, CHU de Clermont-Ferrand, 58 rue Montalembert, Clermont-Ferrand, 63000, France; 3 CHU Clermont-Ferrand, Service Nutrition Clinique, Clermont-Ferrand, France

**Keywords:** Endocannabinoids, Cannabidiol, Skeletal muscle, Atrophy, Sarcopenia, Cachexia, Mitochondria

## Abstract

The endocannabinoid (EC) system is a complex network comprising endogenous ligands, enzymes responsible for their synthesis and degradation, and various receptors (including CB1 and CB2). Present in many peripheral tissues, including skeletal muscle, EC system is now recognized to influence key physiological processes such as insulin sensitivity, mitochondrial metabolism, protein homeostasis and muscle development. Alterations in this system are associated with a variety of pathologies, including obesity, type 2 diabetes, sarcopenia, cachexia and muscle dystrophies. In this context, cannabidiol (CBD), a phytocannabinoid devoid of psychoactive properties, is attracting growing interest as a potential therapeutic agent. This article provides an analysis of the mechanisms by which the EC system, and more specifically the CB1 receptor, influences skeletal muscle development and function, while exploring emerging data on the potential benefits of CBD in various pathological conditions affecting skeletal muscle.

## Graphical abstract

ECS composition and regulation: The endocannabinoid system (ECS) is composed of endogenous ligands (AEA, 2-AG), enzymes for their synthesis or degradation, and receptors (e.g., CB1, CB2). It also includes exogenous molecules like cannabidiol (CBD) produced from *Cannabis sativa*. Widely expressed in peripheral tissues such, the ECS plays a central role in the regulation of key skeletal muscle physiological processes, including insulin sensitivity, mitochondrial metabolism, protein homeostasis and skeletal muscle development. Dysregulation of this system is associated with the development of metabolic and muscular disorders, such as obesity, type 2 diabetes, sarcopenia, cachexia and muscular dystrophies. In this context, CBD, a non-psychoactive phytocannabinoid, has emerged as a potential therapeutic agent capable of modulating ECS activity, thereby contributing to the restoration of skeletal muscle function and homeostasis. ECS: endocannabinoid system, AEA: anandamine, 2-AG: 2-arachidonoyl glycerol, CB1/2: cannabinoid receptor type 1/2, CBD: cannabidiol.

**Figure:**
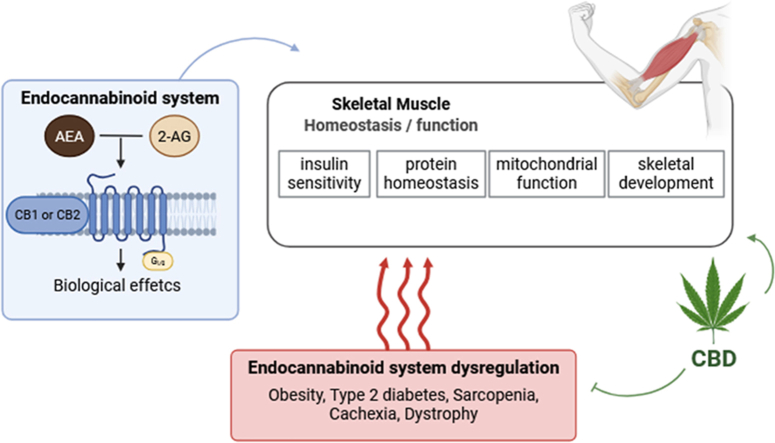

## 1 Introduction

The endocannabinoid system (ECS), present in most mammalian cells, comprises endogenous ligands known as endocannabinoids (eCBs), their enzymatic synthesis and degradation machinery, and at least two specific receptors: CB1 and CB2. These G protein-coupled receptors (Gi/o type) modulate ion channels and activate various signaling pathways. The CB1 receptor is highly expressed in the central nervous system, particularly in neurons regulating food intake and energy expenditure, as well as in peripheral tissues such as skeletal muscle, adipose tissue, and the liver [1,2]. The CB2 receptor is primarily expressed in immune cells, where it plays a key role in inflammatory and immune responses [3], and studies have also observed its expression in peripheral tissues [[4], [5], [6]]. This system also includes exogenous molecules called phytocannabinoids, produced by the *Cannabis sativa* plant, with Δ9-tetrahydrocannabinol (THC) being the most well-known for its psychoactive properties, and cannabidiol (CBD), which lacks such effects [7]. Additionally, a wide range of synthetic cannabinoids can interact with this system, including agonists such as arachidonyl-2′-chloroethylamide (ACEA) and antagonists like rimonabant [8]. This molecular system plays a crucial role in energy homeostasis [9]. It is widely expressed in the brain, particularly in regions involved in the regulation of energy metabolism, such as the hypothalamus, brainstem, and corticolimbic system. The ECS is also present in various peripheral organs, including the liver, adipose tissue, and skeletal muscle, where it exerts significant metabolic functions.

eCBs are synthesized from cellular lipids. Initially described in the central nervous system, they are not stored in presynaptic vesicles (unlike classical neurotransmitters) but are produced on demand in response to increased intracellular calcium levels [2]. Due to their lipophilic nature and rapid degradation, eCBs have a very short half-life and duration of action, and primarily exert autocrine and paracrine effects. The main eCBs are anandamide (AEA) and 2-arachidonoylglycerol (2-AG). AEA and 2-AG belong to the N-acylethanolamines (NAEs) and 2-acylglycerol families, respectively. AEA is synthesized from arachidonoyl-phosphatidylethanolamine (NAPE), which is formed by the transfer of arachidonic acid from a phospholipid to N-phosphatidylethanolamine via N-acetyltransferase (NAT). Hydrolysis of NAPE by NAPE-hydrolyzing phospholipase D (NAPE-PLD) yields AEA, which is subsequently degraded by fatty acid amide hydrolase (FAAH). The synthesis of 2-AG requires activation of phosphoinositol-phospholipase C, which hydrolyzes phosphatidylinositol to diacylglycerol (DAG). DAG is then hydrolyzed by diacylglycerol lipase (DAGL) to form 2-AG, whose degradation is mediated by monoacylglycerol lipase (MAGL) [10]. AEA preferentially binds to the CB1 receptor but can also interact with the CB2 receptor, albeit with lower affinity. In contrast, 2-AG is capable of binding and activating both CB1 and CB2 receptors with similar affinity and greater efficacy than AEA [11].

The aim of this review is to examine the role of the ECS in skeletal muscle physiology, with a particular focus on the distinct and complementary roles of CB1 and CB2 receptors, as well as the therapeutic potential of CBD. We synthesize evidence linking the ECS to the development of anabolic resistance, mitochondrial dysfunction, and inflammatory processes in skeletal muscle under several pathophysiological conditions. While CB1 has been extensively studied for its metabolic and mitochondrial effects, CB2 has recently emerged as a critical regulator of immune-mediated muscle repair, metabolic homeostasis, and regenerative processes. Additionally, we explore how the ECS, and CBD in particular, modulates broader processes and signaling pathways, such as mitochondrial activity, that influence protein anabolism and are essential for skeletal muscle health. Together, these elements provide complementary insights into the ECS mechanisms governing muscle integrity and pathology, while highlighting CBD as a promising non-psychoactive phytocannabinoid for therapeutic applications. For a broader overview of the ECS in peripheral tissues, readers are referred to comprehensive reviews [1,2,9,[12], [13], [14]].

## 2 Role of the endocannbinoid system in skeletal muscle development and function

The ECS plays a fundamental role in skeletal muscle development and the regulation of its contractile properties. During the differentiation of myoblasts into myotubes, 2-AG levels decrease, and *in vitro* treatment with 2-AG or CB1-specific agonists prevents myotube formation. These inhibitory effects are attenuated by CB1 antagonists or CB1 knockdown [15]. *In vivo*, muscle bundles from CB1−/− mouse embryos contain a greater number of muscle fibers, which exhibit a larger diameter after birth compared to wild-type mice [15]. The inhibition of muscle differentiation by 2-AG involves suppression of voltage-dependent Kv7.4 channel activity, which has a permissive role in myogenesis. Conversely, CB1 agonists promote myoblast proliferation [15]. Thus, the EC system may play a key role in the onset of muscular dystrophies or skeletal muscle atrophy by regulating the balance between myoblast proliferation and differentiation into myotubes.

Several studies have reported alterations in CB1 receptor expression in the skeletal muscle of old rodents [16,17], as well as in response to atrophy induced by prolonged fasting [18], statin treatment [19], or cancer [20] in animal models. Similarly, major changes in the expression of enzymes involved in eCB synthesis and degradation have been observed in the skeletal muscle of rodents in the context of sarcopenia [17] or cancer cachexia [20]. CB1 receptor expression is also upregulated in the skeletal muscle and satellite cells of mdx mice (a model of Duchenne muscular dystrophy, DMD), as well as in muscle biopsies from DMD patients [21]. This increase in CB1 expression is regulated by PAX7, a transcription factor involved in the activation and renewal of satellite cells [22]. *In vitro*, CB1 inhibition by rimonabant reduces satellite cell proliferation and promotes their differentiation, while CB1 activation by 2-AG or ACEA has the opposite effect. *In vivo*, rimonabant treatment in mdx mice enhances muscle regeneration, reduces inflammation, and improves locomotor activity [21]. Finally, a recent study demonstrated that muscle-specific deletion of CB1 has a protective effect against the development of insulin resistance and obesity, and controls multiple skeletal muscle processes [23]. Specifically, muscle-specific CB1 deletion promotes myogenesis, leading to increased muscle mass. It also reduces ectopic lipid accumulation in skeletal muscle in response to an obesogenic diet by stimulating mitochondrial activity [23].

The ECS and its associated alterations also influence skeletal muscle functional properties. Physical activity and muscle strength in CB1−/− mice are lower than in wild-type counterparts, and CB1 activation reduces calcium sensitivity of the contractile apparatus, and increases skeletal muscle fatigability [24]. In another study using skmCB1-KD mice (conditional, tamoxifen-induced CB1 inhibition in skeletal muscle), Singlar et al. observed decreased contractile force and increased muscle fatigability [25]. Significant differences in the architecture of the skeletal muscle mitochondrial network were also noted in these mice [25]. The differing responses between the two models may be explained by neuronal effects. CB1 receptors are highly expressed at both pre- and post-synaptic sites in neurons [26]. In CB1−/− mice, global CB1 inhibition, including within the central nervous system, may disrupt neurophysiological mechanisms involved in the regulation of muscle fatigability. In contrast, in skmCB1-KD mice, where CB1 inhibition is muscle-specific and spares neuromuscular junction neurons, neuronal regulation remains intact, potentially accounting for the distinct fatigue resistance phenotype.

## 3 The endocannabinoid system as modulator of the action of insulin in skeletal muscle

Insulin resistance plays a key role in the onset of skeletal muscle wasting [27]. Several studies have demonstrated that insulin resistance can be induced by high-fat diets or obesity, and is associated with elevated plasma concentrations of AEA and 2-AG [[28], [29], [30]], as well as increased tissue levels, particularly in visceral adipose tissue [5,28,30,31]. In obese patients, the amount of visceral adipose tissue is positively correlated with plasma levels of AEA and 2-AG [32]. Other studies have shown that eCB production is regulated by insulin, which stimulates the expression of degradative enzymes (FAAH and MAGL) in adipocytes, thereby reducing their intracellular accumulation [28]. However, this insulin action is abolished in insulin-resistant 3T3-L1 adipocytes [28]. In a recent elegant study, Yang et al. demonstrated a significant link between the development of whole-body insulin resistance induced by high levels of endocannabinoids (eCBs) and the secretion of resistin by peripheral blood mononuclear cells (PBMC) [33]. The authors first showed *in vitro* that 2-AG increased resistin production by PBMC, an effect that was inhibited by rimonabant, a CB1 antagonist. Notably, resistin was produced exclusively in monocytes expressing CB1. In a high fat diet preclinical model, they further demonstrated that high levels of 2-AG led to the migration of CB1-receptor-expressing monocytes into adipose tissue, an effect associated with the development of insulin resistance and glucose intolerance. These metabolic disturbances were abolished in resistin KO mice, highlighting the critical role of resistin and immune system in mediating eCB-induced insulin resistance [33]. Thus, elevated circulating levels of eCBs may not only result from insulin resistance but also exacerbate its effects by promoting visceral adipose tissue expansion, establishing a deleterious metabolic vicious cycle for skeletal muscle.

Skeletal muscle plays a pivotal role in the regulation of energy homeostasis, not only during periods of high activity requiring substantial energy expenditure but also at rest, making it a major site of glucose utilization. Numerous studies have confirmed the presence of CB1 and CB2 receptors, as well as many components of the ECS, in rodent and human skeletal muscle, and in various muscle cell models [6,16,[34], [35], [36], [37]]. The expression of CB1 and CB2 receptors and eCB production in skeletal muscle are influenced by obesity. Specifically, CB2 expression is decreased in the abdominal wall muscle of rats fed a high-fat diet [38], whereas CB1 expression remains unchanged in myotubes from healthy or obese subjects [39]. Similarly, in Zucker rats, CB1 expression in the *soleus* muscle is reduced, while AEA content is increased [40,41]. Conversely, CB1 expression is elevated in the *soleus* of mice fed a high-fat diet, with increased 2-AG levels but no change in AEA [42,43]. These findings suggest that the ECS's response to the organism's metabolic state may depend on diet, species, and muscle type.

Pharmacological studies have demonstrated that inhibition of CB1 activity in rodents and obese patients increases oxygen consumption and energy expenditure, associated with enhanced fatty acid oxidation and mitochondrial biogenesis [[44], [45], [46]]. Furthermore, chronic treatment with the CB1 antagonist rimonabant in *ob/ob* mice and Zucker rats, whether obese or lean, improves glucose uptake in the *soleus* muscle [40,44]. Conversely, CB1 activation via injection of the agonist HU210 reduces glucose uptake. These effects have been reproduced *in vitro*. Specifically, incubation of muscle cells with CB1 agonist AEA is sufficient to decrease glucose uptake, an effect that is inhibited by the CB1 antagonists, i.e. rimonabant or AM251 [36]. In contrast, CB1 inhibition by rimonabant alone or via genetic knockdown using siRNA stimulates glucose absorption [39].

Regulation of glucose uptake by CB1 involves the PI3K/Akt pathway. Prolonged treatment of myotubes with rimonabant increases intracellular content of both the regulatory (p85α) and catalytic (p110α) subunits of PI3K [39]. This is accompanied by enhanced Akt phosphorylation and glucose uptake, an effect inhibited by the PI3K inhibitor LY294002 [39]. Conversely, incubation of human muscle cells with AEA disrupts Akt phosphorylation [36]. While the link between the ECS and insulin resistance is widely accepted, some studies have not observed changes in Akt phosphorylation in response to rimonabant or WIN 55,212-2 (a mixed CB1/CB2 agonist) [40,47]. Therefore, the precise conditions (dose, exposure duration) and stimuli (pharmacological CB1 antagonists/agonists, eCBs) that modulate this signaling pathway require further investigation. Another key player in insulin action is the MAPK/ERK pathway, which is involved in the control of cell proliferation and gene expression regulation. CB1 appears to interact with this pathway, but results are contradictory: the CB1 agonist ACEA inhibits insulin-induced ERK phosphorylation [47], whereas the CB1 agonist AEA stimulates it [36]. Taken together, these data suggest that activation of the ECS in muscle alters insulin signaling and glucose uptake. This has a direct impact on protein homeostasis, particularly protein synthesis, which is stimulated by anabolic hormones such as insulin. Indeed, treatment of cultured myotubes with rimonabant increases Akt phosphorylation and stimulates protein synthesis in a dose- and time-dependent manner [48]. Another fundamental aspect of metabolism, closely linked to insulin action and protein homeostasis, is mitochondrial function. This warrants particular attention, as it also appears to be regulated by the ECS.

## 4 The endocannabinoid system and mitochondrial function in skeletal muscle

A study by Mendizabal-Zubiaga et al. revealed that nearly 25% of skeletal muscle mitochondria express the CB1 receptor, and that ∼60% of CB1 receptors present in skeletal muscle are localized at the mitochondrial level [34]. These findings suggest a modulatory role of the ECS on mitochondrial activity and biogenesis. Treatment of cells or isolated mitochondria with CB1 agonists leads to impaired mitochondrial integrity, respiratory activity, ATP production, and mitochondrial biogenesis [46,[49], [50], [51]]. eCBs also influence mitochondrial morphology and respiration. For example, the CB1 agonist AEA induces mitochondrial swelling, accompanied by a decrease in membrane potential and alterations in membrane permeability [49,52,53]. Conversely, inactivation of the ECS stimulates mitochondrial biogenesis, β-oxidation, and the activity of Krebs cycle [44,46,[54], [55], [56]]. These results are also observed *in vivo*. A recent study in mice showed that specific deletion of the CB1 gene in skeletal muscle (mCB1−/−) led to increased muscle mass, mitochondrial biogenesis, maximal mitochondrial oxidative capacity, and ATP-coupled respiration [23]. This suggests that muscle mitochondria in mCB1−/− mice are more efficient in energy production. Similarly, whole-body CB1 knockout in mice (CB1−/−) enhances oxidative capacity in skeletal muscle, as evidenced by increased activity of complexes I and IV, and is accompanied by elevated radical oxygen species (ROS) production [57], indicating greater oxidative stress. However, a recent study by Singlar et al. introduced a mouse model with a global, mitochondria-specific deletion of the CB1 gene (mtCB1−/−) [58]. Unlike previous findings in CB1−/− and mCB1−/− models, this study revealed striking mitochondrial abnormalities [58]. Specifically, mtCB1−/− mice exhibited morphological defects, including swollen and irregularly shaped mitochondria, alongside impaired mitochondrial respiration. These dysfunctions were characterized by reduced oxidative phosphorylation and diminished electron transport system capacity [58]. These findings suggest that the subcellular localization of the CB1 receptor may underlie its differential effects on mitochondrial physiology. However, further studies are required to elucidate the molecular mechanisms involved and to reconcile the apparent discrepancies observed between mitochondrial-specific knockout models (mtCB1−/−) and whole-cell or tissue-specific CB1 deletion models (CB1−/− and mCB1−/−).

The absence of CB1 also alters mitochondrial dynamic, which is essential for adjusting the quantity and quality of mitochondria according to cellular needs. For instance, both studies reported increased expression of PGC1α (PPARG coactivator 1 alpha) and TFAM (transcription factor A, mitochondrial) transcripts, two transcription factors critical for mitochondrial biogenesis [23,57]. Furthermore, increased expression of OPA1 (OPA1 mitochondrial dynamin like GTPase, modulating mitochondrial fusion) and Parkin (modulating mitophagy) transcripts suggests an adaptation of mitochondrial dynamic to eliminate damaged mitochondria and promote mitochondrial fusion to meet energy demands [57]. The increase in oxidative stress, mitochondrial respiration, and muscle mass in the absence of CB1 may seem contradictory, but these phenomena can be linked in the context of cellular adaptation and skeletal muscle physiology. The observed increase in mitochondrial respiration may reflect an adaptation to heightened energy demand associated with increased skeletal muscle mass. Although often linked to cellular damage, elevated ROS production may act as a cellular stimulus promoting skeletal muscle growth and adaptation. One mechanism potentially explaining the deleterious effects of the ECS on mitochondrial integrity and function could be the production of toxic lipid intermediates, such as ceramides [59,60]. For example, a recent study revealed that CB1 inactivation by injection of JD5037 (a CB1 inverse agonist) restored insulin sensitivity in a model of high-fat diet-induced obesity by reducing *de novo* ceramide synthesis in the liver [60].

## 5 The multifaceted role OF CB2 receptor in skeletal muscle: integrating metabolism and immunity

Cannabinoid type 2 receptor (CB2) has emerged as critical regulator of skeletal muscle homeostasis, integrating immune modulation, metabolic control, and regenerative processes. CB2 is expressed in both human and rodent skeletal muscle, as well as in muscle-resident immune cells such as macrophages, where it contributes to the local ECS alongside CB1 and TRPV1 [6,15,61]. Its expression is dynamically regulated in response to injury, with a marked upregulation in infiltrating polymorphonuclear cells and macrophages during muscle healing, co-localizing with macrophage markers and suggesting a central role in immune-mediated repair [62]. In ischemia–reperfusion injury models, activation of CB2 receptors attenuates oxidative damage and promotes early myogenesis via the Nrf2 signaling pathway [63], whereas CB2 deficiency impairs myofiber regeneration [64], underscoring its protective role in muscle recovery. A key mechanism underlying these effects is the regulation of macrophage polarization. CB2 signaling modulates macrophage polarization, favoring an anti-inflammatory M2 phenotype that enhances myoblast differentiation and tissue regeneration, while its absence promotes a pro-inflammatory M1 phenotype that exacerbates inflammation and impairs skeletal muscle repair [61,64]. This immune-modulatory function is further supported in inflammatory conditions such as DMD, where CB2 activation reduces pro-inflammatory cytokine production and promotes M2 polarization [65]. Beyond its role in immune regulation, CB2 is predominantly expressed on immune cells, including macrophages, neutrophils, and lymphocytes, where its activation generally suppresses inflammatory responses, while its absence amplifies cytokine production and tissue damage [66]. In skeletal muscle contusion models, CB2 agonism ((e.g., JWH-133) reduces inflammatory cell infiltration and lowers levels of pro-inflammatory (TNF-α, IL-1β, IL-6) and pro-fibrotic mediators (TGF-β, IL-4, IL-13), while increasing IL-10 [62,67,68]. This immune modulation translates into reduced fibrosis, diminished collagen deposition, and enhanced formation of regenerating myofibers.

In addition to its immunomodulatory effects, CB2 directly influences skeletal muscle metabolism. Activation by agonists such as β-caryophyllene enhances glucose uptake, stimulates glycolytic and oxidative pathways, increases ATP production, and promotes lipid oxidation via SIRT1/PGC-1α signaling in myotubes [69]. These metabolic effects align with mechanistic evidence identifying CB2 as a positive regulator of energy metabolism, muscle cell growth, and regeneration [61]. Collectively, current evidence demonstrates that CB2 integrates immune and metabolic signaling to limit inflammation and fibrosis while promoting efficient muscle repair. These findings highlight its potential as a therapeutic target for muscle disorders characterized by chronic inflammation, degeneration, and metabolic dysfunction.

## 6 Cannabidiol as a regulator of skeletal muscle integrity

The potential of cannabidiol (CBD) has been investigated in various cardiovascular, neurodegenerative, oncological, and metabolic pathologies [70]. More recently, a growing number of studies have explored its impact on skeletal muscle development, regeneration, metabolism, inflammation, and protein anabolism.

The development of obesity is accompanied by ectopic lipid accumulation in peripheral tissues, such as skeletal muscle [71]. This phenomenon, known as lipotoxicity, contributes to the onset of insulin resistance. A series of studies conducted by Bielawiec et al. in rats fed with an obesogenic diet highlighted the protective effects of CBD against insulin resistance and lipotoxicity [[72], [73], [74]]. In this context, chronic CBD treatment reduced intramuscular fatty acid accumulation and inhibited *de novo* lipogenesis [72], prevented oxidative stress, and attenuated inflammation [74]. Additionally, CBD limited obesity-induced ceramide synthesis (a deleterious lipid derivative), which coincided with improved insulin sensitivity [73]. Beyond its protective role against lipotoxicity and inflammation, CBD may also play a key role in preserving and regenerating muscle tissue.

Skeletal muscle dystrophies represent a heterogeneous group of hereditary diseases characterized by progressive and irreversible skeletal muscle tissue damage. Among these, Duchenne muscular dystrophy (DMD), caused by dystrophin deficiency, leads to a reduced capacity for muscle regeneration, particularly affecting satellite cells [75]. Building on the role of the ECS in skeletal muscle development control [15], Iannotti et al. explored the use of CBD as a treatment in a DMD model (mdx mice) [76]. The beneficial effects of CBD had functional repercussions, as the locomotor abilities were improved in mdx mice treated with CBD for 2 weeks [76]. Furthermore, this work demonstrated that CBD stimulated the differentiation of human myoblasts and restored the differentiation capacity of human myoblasts or satellite cells isolated from DMD patients [76].

In addition, CBD has shown protective effects in models of acute muscle injury. In treadmill-induced muscle injury, CBD preserved muscle structure, reduced inflammatory cell infiltration, and normalized serum markers of muscle damage such as creatine kinase (CK) and lactate dehydrogenase (LDH), as well as, at the systemic level, the cortisol/testosterone ratio [77]. CBD also improved antioxidant status by decreasing malondialdehyde (MDA) levels and increasing superoxide dismutase (SOD) and glutathione peroxidase (GSH-Px) activities [77]. Mechanistically, CBD downregulated pro-inflammatory and oxidative signaling pathways (IL-6, TNF-α, NF-κB, Keap1) and mitophagy-related proteins (AMPKα2, HIF-1α, BNIP3, NIX), while upregulating the antioxidant response via Nrf2/HO-1 and the anti-inflammatory cytokine IL-10 [77]. These findings suggest that CBD may exert its protective effects on skeletal muscle through a combination of anti-inflammatory, antioxidant, and mitochondrial regulatory mechanisms.

Given the properties described above, it has been shown that CBD can help to limit the loss of muscle mass and function in certain pathophysiological conditions. Cachexia is a multifactorial wasting syndrome characterized by weight loss and skeletal muscle mass wasting, sometimes accompanied by a reduction in fat mass. This syndrome manifests in numerous pathologies, including heart failure, AIDS, and cancer [78]. Cancer treatment often involves chemotherapy, which itself contributes to muscle atrophy [79]. In a recent study, cultured myotubes were exposed to conditions mimicking chemotherapy treatment (cisplatin), and the effects of CBD on various parameters, including atrophy, protein homeostasis, oxidative stress, and cell viability, were evaluated. Cisplatin induced myotube atrophy, associated with reduced cell viability and increased apoptosis. Under these conditions, the addition of CBD prevented cellular atrophy and reduced cell death [80]. This study showed that cisplatin-induced muscle atrophy primarily resulted from dysregulation of protein homeostasis (decreased protein synthesis and increased proteolysis), partly caused by oxidative stress. CBD restored protein synthesis and reduced proteolysis. These anti-atrophic effects of CBD could be partly attributed to its antioxidant activity, as evidenced by the modulation of TBARS content (a marker of lipid peroxidation), catalase activity, and the expression of mRNA for various antioxidant systems [80]. Cisplatin-induced oxidative stress in cultured myotubes was also associated with major alterations in the expression of proteins involved in the mitochondrial respiratory chain and ATP production, disruptions that were corrected by CBD [80]. Similar to chemotherapy-induced skeletal muscle atrophy, disuse-related skeletal muscle atrophy is also strongly associated with inflammation and oxidative stress [81]. In a recent study, Kato et al. used a sciatic nerve resection model to induce skeletal muscle atrophy. They observed that two weeks of CBD administration via gavage helped to mitigate the loss of skeletal muscle mass and the decline in running capacity [82]. Additionally, CBD reduced oxidative stress and skeletal muscle inflammation, as evidenced by decreased TNFα expression, and suppressed the denervation-induced upregulation of atrophy-related genes, such as atrogin-1 and MuRF1 [82].

Moreover, several studies have demonstrated that CBD can modulate mitochondrial activity. In rats, intense exercise leads to a decrease in mitochondrial membrane potential and a reduction in muscle ATP content. These effects were generally attenuated by CBD [83]. Similarly, doxorubicin treatment, which induces cardiomyopathy in mice, was characterized by increased oxidative stress and apoptosis in cardiac tissue, mirroring the results observed after cisplatin treatment in myotubes. Doxorubicin also reduced mitochondrial biogenesis and activity [84]. In this model, CBD administration limited oxidative stress while improving mitochondrial function and biogenesis [84]. In another rat model submitted to intense-exercise, CBD improved mitochondrial structure and function, and downregulated key mitophagy markers such as PINK1, Parkin, and BNIP3, indicating an inhibition of excessive mitophagy and a preservation of mitochondrial integrity in skeletal muscle [83]. Lastly, in a disuse-induced skeletal muscle atrophy model, where PGC1α mRNA expression, mitochondrial DNA, and ATP levels were reduced, CBD administration attenuated these decreases [82]. Thus, the protective effects of CBD on muscle protein homeostasis may result from its action on mitochondrial activity and, therefore, ATP production, which is essential for protein synthesis.

## 7 Cannabidiol as a regulator of inflammation and immune response in skeletal muscle

CBD has demonstrated clear immunomodulatory and anti-inflammatory effects across numerous models, primarily influencing skeletal muscle by reducing inflammation and oxidative stress. However, direct evidence linking immune changes to functional muscle outcomes in humans remains limited and is largely derived from preclinical studies.

At the molecular level, CBD exerts broad immunosuppressive and immunomodulatory effects. It directly inhibits the activation of several types of immune cells, induces apoptosis, and promotes the proliferation of regulatory cells, such as myeloid-derived suppressor cells, which control the immune response (for review, see [85]). CBD also suppresses key pro-inflammatory cytokines, including TNF-α, IL-1β, IL-6, and IFN-γ, while modulating transcription factors such as NF-κB [[86], [87], [88]]. In macrophages and monocytes, CBD decreases LPS-induced NF-κB activity and reduces the release of IL-8 and MCP-1 [88,89]. Furthermore, CBD alters the production of lipid mediator in macrophages from pro-inflammatory eicosanoids toward specialized pro-resolving mediators, thereby promoting the resolution of inflammation [90].

Although CBD has low direct affinity for CB1 and CB2 receptors, and many of its effects persist in CB1/CB2 knockout mice, some immunomodulatory actions of CBD can be attenuated or reversed by CB2 antagonists, indicating a partial CB2 dependence [91]. A key example of direct CBD interaction with CB2 in immunity comes from a murine model of acute graft-versus-host disease, where CBD increased CB2 expression on CD4^+^ and FoxP3^+^ regulatory T lymphocytes, reduced pro-inflammatory chemokines and cytokines, and improved survival [92]. These effects were partially lost when CB2 was blocked, demonstrating functional dependence on CB2 [92]. Similarly, in human T lymphocytes, a CBD-rich extract inhibited proliferation and cytokine production, effects that were partly reversed by a CB2 antagonist, further implicating CB2 in these processes [93].

In skeletal muscle, a narrative review reports that CBD reduces inflammation following eccentric exercise, muscular dystrophy, and obesity by lowering NF-κB, IL-6, TNF-α, and COX-2 levels [94]. In exercise-induced skeletal muscle injury in rats, CBD reduced inflammatory cell infiltration and downregulated IL-6, TNF-α, and NF-κB, while increasing IL-10 and Nrf2/HO-1, suggesting protection via inhibition of oxidative stress and inflammation [77]. Furthermore, in dystrophic muscle, CBD improved satellite cell differentiation, indicating potential support for muscle regeneration under chronic inflammatory conditions [76].

## 8 Human evidence on cannabidiol for exercise recovery and performance

The potential recovery-promoting effects of CBD in physically active populations have been explored across a range of exercise modalities, although the current evidence remains inconclusive. To our knowledge, only one study by van Doorslaer de Ten Ryen et al. has investigated the link between exercise and the ECS in humans [95]. In this study, which combined resistance and endurance exercise, the authors demonstrated that the ECS is dynamically modulated by exercise. They also showed that the exercise modality impacts ECS activity, with resistance and endurance exercise regulating the levels of eCBs and CB1 expression in opposite ways [95]. Given the impact of exercise on the ECS, exploring the effect of ECS modulators, such as CBD, could lead to a better understanding of their potential to improve skeletal muscle recovery or performance. In trained athletes exposed to a 6-day high-intensity resistance training protocol, oral CBD supplementation (60 mg) did not improve performance-related outcomes, such as squat and bench press performance, or 1-mile running time [96]. Similarly, no clear anti-inflammatory effect was observed, as indicated by the absence of significant changes in circulating IL-6, IL-10, or blood immune cell ratios [96]. Although CBD oil was associated with a modest reduction in post-exercise myoglobin concentrations in “advanced” athletes, this effect was not evident in “highly advanced” individuals. Moreover, blood creatine kinase (CK) concentrations and performance variables remained unchanged, leading the authors to conclude that the effects of CBD on exercise-induced muscle damage and performance are, at present, equivocal [96].

Evidence from both untrained and trained subjects further questions the practical utility of CBD for exercise recovery and performance enhancement. In untrained adults, topical CBD application for 3 days after fatiguing isokinetic exercise failed to significantly influence pain sensitivity, neuromuscular performance, or torque production relative to placebo or rest. Moreover, the greater perceived benefit reported in the placebo condition compared with the no-treatment condition suggests that expectancy-related mechanisms may partially explain subjective improvements rather than any specific physiological effect of CBD [97]. In trained runners, acute pre-exercise CBD supplementation (50–300 mg) similarly failed to improve perceptual, affective, or performance outcomes during endurance running. Although small dose-dependent metabolic effects were observed, these were not translated into meaningful benefits for exercise capacity or post-exercise recovery, as reflected by unchanged CK and myoglobin responses [98].

Last, a 2024 systematic review including studies conducted in healthy, physically active individuals, similarly concluded that the current data do not provide sufficient evidence to confirm that CBD has ergogenic effects or consistently promotes recovery. Reported outcomes showed limited or equivocal effects on physiological variables (e.g. VO_2_), strength-related measures, and indices of post-exercise recovery [99]. Importantly, this review also underscored several recurring methodological constraints, including small sample sizes and marked heterogeneity in experimental designs and supplementation protocols, which substantially limit the interpretability and generalizability of the existing literature [99].

Altogether, existing evidence does not currently support CBD as an effective strategy to enhance recovery or performance in physically active populations. Although some modest effects have been reported, these findings remain inconsistent and of limited practical significance. More rigorous and standardized research is therefore needed to determine whether CBD has any meaningful application in exercise and sport recovery.

## 9 Conclusion: key insights and future directions

The ECS emerges as a pivotal regulator of skeletal muscle physiology, orchestrating critical processes such as muscle development (through the modulation of myoblast proliferation and myotube differentiation), metabolic homeostasis (by fine-tuning glucose uptake and insulin sensitivity), protein turnover, and mitochondrial function. Accumulating evidence links ECS dysregulation to the pathogenesis of metabolic disorders (e.g., obesity, type 2 diabetes), degenerative muscle diseases (e.g., Duchenne muscular dystrophy), and skeletal muscle-wasting conditions (e.g., cachexia and sarcopenia).

Pharmacological interventions targeting the ECS, particularly with synthetic cannabinoids, have demonstrated therapeutic potential in restoring physiological balance in preclinical models. Amongst these, emerging evidence point to CBD, a non-psychoactive phytocannabinoid, as a promising therapeutic agent due to its ability to mitigate lipotoxicity, oxidative stress, and inflammation, whilst enhancing skeletal muscle satellite cell function, myoblast differentiation, and protein synthesis in preclinical models. By restoring mitochondrial integrity, modulating inflammation and immune response, and counteracting chemotherapy- or disease-induced atrophy, CBD offers a multifaceted approach to preserving skeletal muscle mass and function. However, despite these promising mechanistic and preclinical findings, current human evidence remains limited and inconsistent, particularly regarding functional outcomes such as exercise recovery and performance. This discrepancy likely reflects the complexity of CBD's action, as more than 75 identified cellular targets have been identified [100]. In addition, its effects may be indirect, mediated via immune cells or systemic factors rather than direct modulation of myotube signaling. In this context, identifying the key molecular targets mediating CBD's protective effects in skeletal muscle is a crucial step towards developing more selective and effective adjuvant therapeutic strategies. Furthermore, while many studies have demonstrated ECS alterations associated with skeletal muscle atrophy, emerging evidence suggests that subcellular localization of ECS components (e.g., mitochondrial vs. cytosolic CB1) may critically influence their activity, as highlighted by phenotypic differences between muscle-CB1^−/−^ and mitochondrial-CB1^−/−^ models [23,58]. Accordingly, future research should prioritize on investigating the mitochondrial ECS and its alterations in physiological and pathological conditions to clarify its role in muscle maintenance. Finally, skeletal muscle regeneration is a highly orchestrated process involving the sequential activation and interaction of multiple cell types, such as satellite cells, macrophages, and endothelial cells [101]. While preliminary studies demonstrated that ECS modulation may enhance skeletal muscle regeneration [21,76,102], the precise alterations of the ECS within each of these cell types throughout the regenerative process remain poorly characterized and require further investigation.

## CRediT authorship contribution statement

**Anaïs Deglos:** Writing – original draft, Formal analysis, Data curation, Conceptualization. **Nicolas Saroul:** Writing – original draft, Conceptualization. **Stéphane Walrand:** Writing – review & editing, Conceptualization. **Olivier Le Bacquer:** Writing – review & editing, Supervision, Funding acquisition, Conceptualization.

## Funding

This research was supported by grants from the French government's National Research Agency through the ‘Investissements d’Avenir’ programme (16-IDEX-0001 CAP 20-25), and the Cancéropôle Lyon Auvergne-Rhône-Alpes (CLARA) through its Emergence programme as part of the Cancéropôle accreditation awarded from 2023 to 2027 by the French 10.13039/100000054National Cancer Institute (10.13039/501100006364INCa) for their support of our work in this field.

## Declaration of competing interest

The authors declare that they have no known competing financial interests or personal relationships that could have appeared to influence the work reported in this paper.

## Data Availability

No data was used for the research described in the article.
